# Photoperiodic manipulation modulates the innate and cell mediated immune functions in the fresh water snake, *Natrix piscator*

**DOI:** 10.1038/s41598-020-71777-2

**Published:** 2020-09-07

**Authors:** Alka Singh, Ramesh Singh, Manish Kumar Tripathi

**Affiliations:** 1 Department of Zoology, Udai Pratap Autonomous College, Varanasi, Uttar Pradesh 221 002 India; 2 Present Address: Department of Zoology, School of Life Sciences, Guru Ghasidas Vishwavidyalaya (A Central University), Bilaspur, Chhattisgarh 495 009 India

**Keywords:** Immunology, Zoology

## Abstract

Objectives of the current work were to investigate the role of photoperiod and melatonin in the alteration of immune responses in a reptilian species. Animals were kept on a regimen of short or long days. Blood was obtained and leucocytes were isolated to study various innate immune responses. Lymphocytes were separated from blood by density gradient centrifugation and were used to study proliferation. Respiratory burst activity was measured through nitrobluetetrazolium reduction assay while nitric oxide production by leucocytes was assayed by nitrite assay. Lymphocytes were isolated and used to study proliferation with and without B and T cell mitogens. Photoperiodic manipulation acted differentially on leucocyte counts. Nitrite release was increased while superoxide production was decreased in cultures obtained from the snakes kept on the short day regimen. Significant enhancement of mitogen induced lymphocyte proliferation was observed in cultures from the animals kept in either long or short days compared to cultures from the animals kept in natural ambient day length. Use of in vitro melatonin showed that lymphocytes from the animals, kept in long days, were more reactive. Photoperiod induces changes in immune status which may permit adaptive functional responses in order to maintain seasonal energetic budgets of the animals. Physiological responses (like elevated immune status) are energetically expensive, therefore, animals have evolved a strategy to reduce immune functions at times when energy is invested in reproductive activities. *Natrix piscator* breeds from September to December and elevated pineal hormone in winter suppresses reproduction while immunity is stimulated.

## Introduction

Seasonal adaptations by organisms tend to reflect interactions and coordinations between changing environmental conditions and individual internal rhythms. It is well known that immune function and reproduction are energetically costly physiological processes hence incompatible simultaneously^1^. Photoperiodic information, which is considered as the most effective initial predictive cue, tends to initiate and terminate seasonal adaptations^2^. Well documented fluctuations in disease and pathogen load are natural threats faced by wild populations^3^. However, rapid adaptations have been documented in wild populations in response to seasonal oscillations in climate and pathogen load^4,5^. The endocrine system has been shown to mediate communication between reproduction and immunity^6^. A variety of environmental factors, such as day length, social interaction, food availability, and temperature, may have significant influence on endocrine system that can change reproductive function and immunity^7,8^. For example, the effects of photoperiodic alteration on immune functions have been well studied in mammals^8–11^. The involvement of melatonin has also been implicated in immune functions via experimental alteration in photoperiod^12–14^. Changes in the immune responses that occur in response to maintaining animals in short days are likely to be directly or indirectly related to elevated melatonin secretion. Along these lines, enhancement of immune function in winter has been studied in a variety of rodent species^15^. Authors of various studies have found fairly robust effect of short-day increase in splenic mass in *Peromyscus maniculatus*^16^ and *Mesocricetus auratus*^17,18^. Among non-mammals, melatonin mediated changes in the immune functions have mainly been described in birds^19^. Some reports in birds demonstrate that photoperiod influences immune function^20–22^. Oxidative stress in leucocytes, measured by reactive oxygen species (ROS) level and lymphocyte proliferation was found to be seasonal^23^. In light of these studies, it may be that changes in the immune responses that occur in response to maintaining animals on short day regimens are likely to be directly or indirectly related to elevated melatonin secretion. To date, studies have shown that the efficacy of a given organism’s immune response can vary seasonally. However, as of yet our understanding of how photoperiod modulates immune functions remains limited. According to the energy trade-off hypothesis, elevated melatonin level in winter suppresses gonadal function while immunity is enhance^24^. Most research in this field is focused on mammals and birds, however, the study of unconventional animal models still represent a useful approach to obtain an insight of how photoperiod mediated immune function has changed during the course of vertebrate evolution. Limited works in lower vertebrates suggest that seasonality affects reproduction, food intake, locomotor activity, growth rate, and immune functions of teleosts^25–27^. Photoperiodic manipulation is utilized to affect sexual maturation and growth rate of many fishes^28^. Information regarding photoperiod induced alteration in immune function is currently unavailable in reptiles, despite the fact that developing a systemic approach to understand elements of immune function across vertebrate groups will ultimately help us to better understand the mammalian immune system. Information on this subject in reptiles is of particular significance from comparative point of view, as reptiles represent a pivotal phylogenic group, intermediate between heterotherms and homeotherms. In addition, certain characteristics make reptiles ideal for immunological research, such as optimal body temperature ranges that, when changed, may accelerate or slow down the immune response and the influence of season that may shut down or activate immune system. The checkered keelback snake (*Natrix piscator*) is an ovipararous reptile, endemic to Asia that breeds seasonally. In seasonal breeders the length of melatonin secretion at night appears to have a direct effect on the seasonal variation of physiological processes^29^. We have recently reported the variation in various immune functions due to changing environment in this ophidian species^30^ where daily and seasonal rhythms in immune responses were found. Along with circadian rhythms, we have reported significant circannual rhythm in seven out of nine tested immune parameters. In a previous study, we have also demonstrated that *N. piscator* exhibits seasonal variation in blood immunological traits^31^. Based on this research, we hypothesized that photoperiod plays an important role in the regulation of immune responses in this species. Specifically, we investigated the role of melatonin in modulation of lymphocyte proliferation as a method to deduce a relationship between the immune and endocrine systems. Understanding the immunity and its interaction with seasonality has provided important insights in the evolution of this physiological process.

## Results

**Figure 1:**
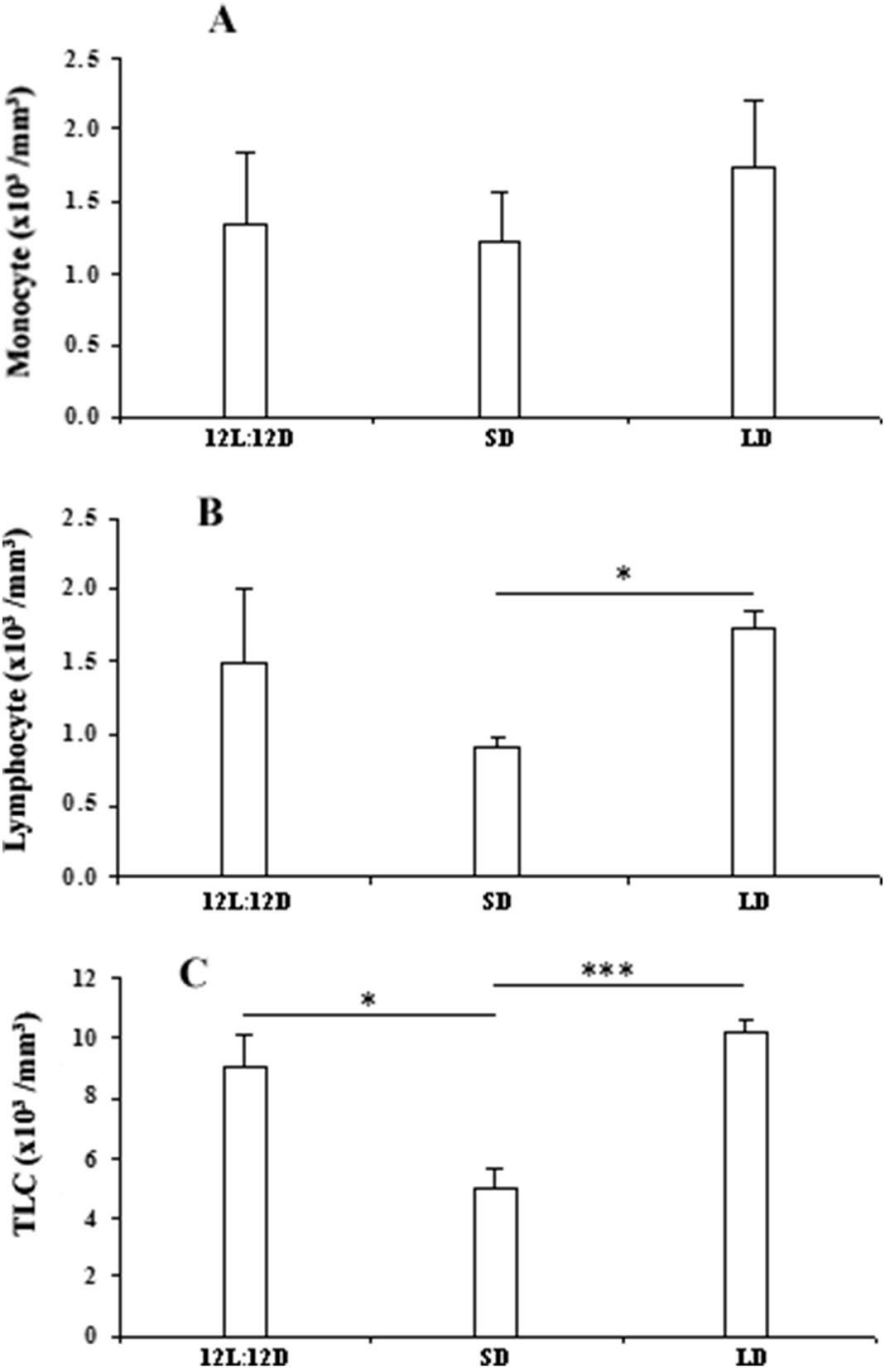
Effect of different photoperiodic regimens (12L:12D—light:dark 12:12; SD—short days, light:dark 8:16; LD—long days, light:dark 16:8) on total leucocytes count (TLC) **(C)**, lymphocyte **(B)** and monocyte **(A)** count in the fresh-water snake, *Natrix piscator*. Data were analysed by One Way ANOVA: * (p < 0.05); *** (p < 0.001).

**Figure 2:**
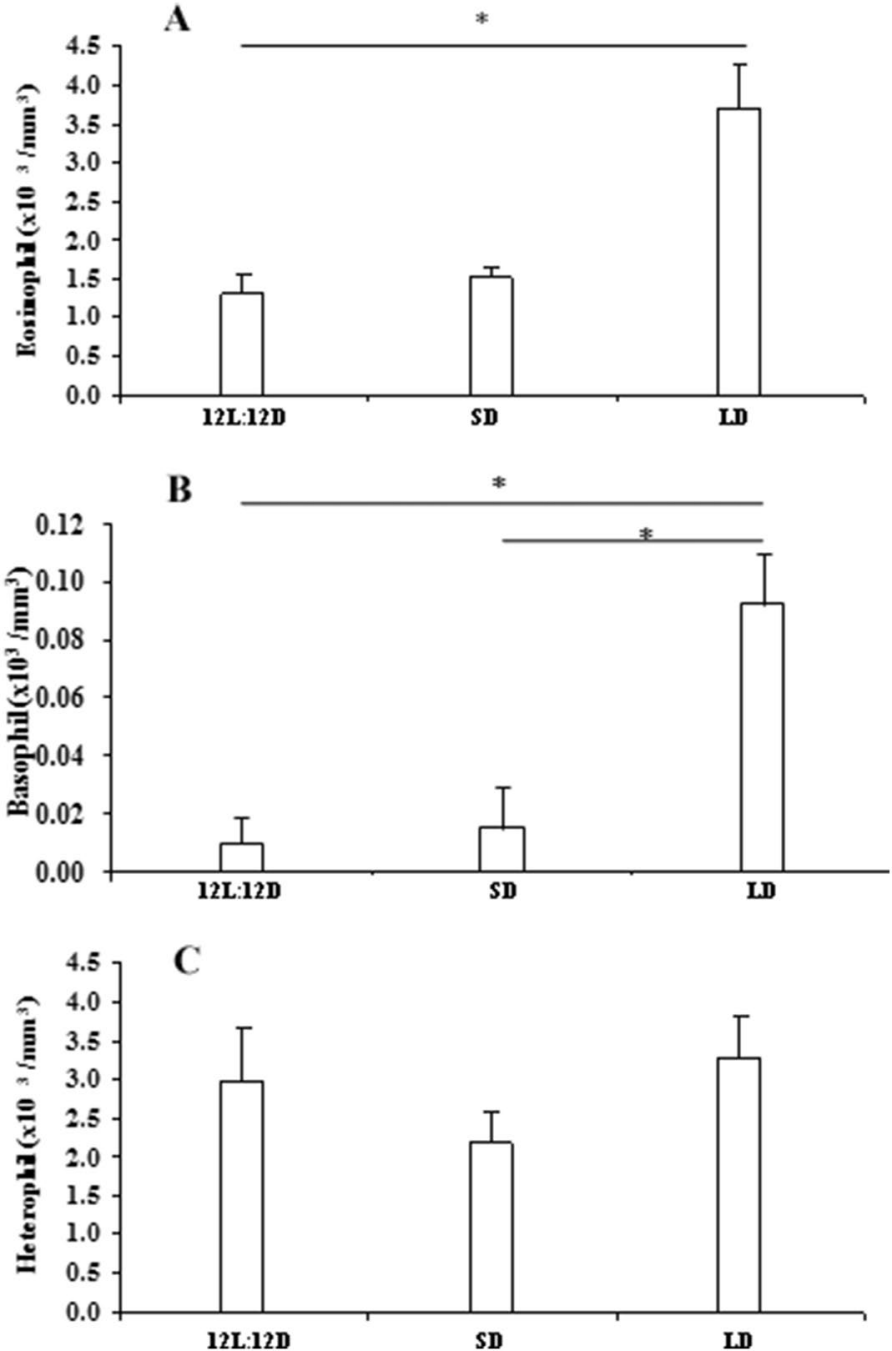
Effect of different photoperiodic regimens (12L:12D—light:dark 12:12; SD—short days, light:dark 8:16; LD—long days, light:dark 16:8) on eosinophil **(A)**, basophil **(B)** and heterophil **(C)** count in the fresh-water snake, *Natrix piscator*. Data were analysed by One Way ANOVA: * (p < 0.05); *** (p < 0.001).

No significant change was observed in the number of monocyte due to photoperiodic manipulation (Fig. 1A). Total leucocytes decreased significantly (df = 14; F = 17.862 and p < 0.001) in snakes subjected to short days in comparison with snakes maintained in natural day length (12L:12D) or long days (Fig. 1C). Lymphocyte count also decreased (df = 14; F = 4.971 and p < 0.05) in the blood of animals kept in short days when compared with the animals kept in long days (Fig. 1B). Significant increase in the number of basophil (df = 14; F = 32.817 and p < 0.001) (Fig. 2B) was observed in the snakes kept in long days, when compared to the snakes maintained either in natural day length or in short days. Eosinophil count was also increased (df = 14; F = 38.924 and p < 0.001) in long day animals as compared to animals kept in natural day length (Fig. 2A).

**Figure 3:**
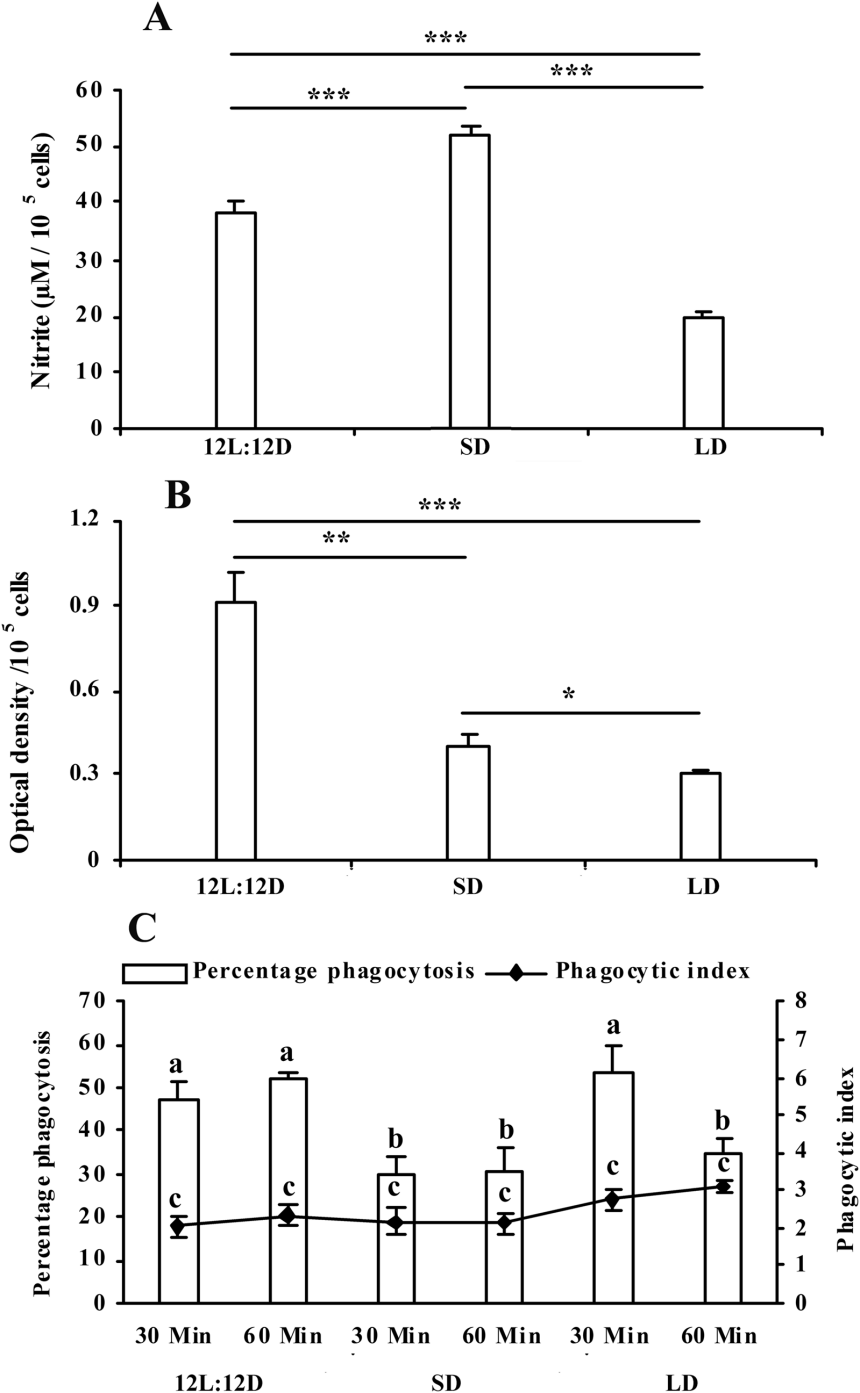
Effect of different photoperiodic regimens (12L:12D—light:dark 12:12; SD—short days, light:dark 8:16; *LD—*long days, light:dark 16:8) on leucocyte phagocytosis **(C)**, superoxide production **(B)** and nitrite release **(A)** in the fresh-water snake, *Natrix piscator*. Data were analysed by One Way ANOVA and Post hoc comparisons were done utilizing Newman-Keul’s multiple-range test* (p < 0.05); *** (p < 0.001). Error bars bearing same superscript do not differ significantly.

**Figure 4:**
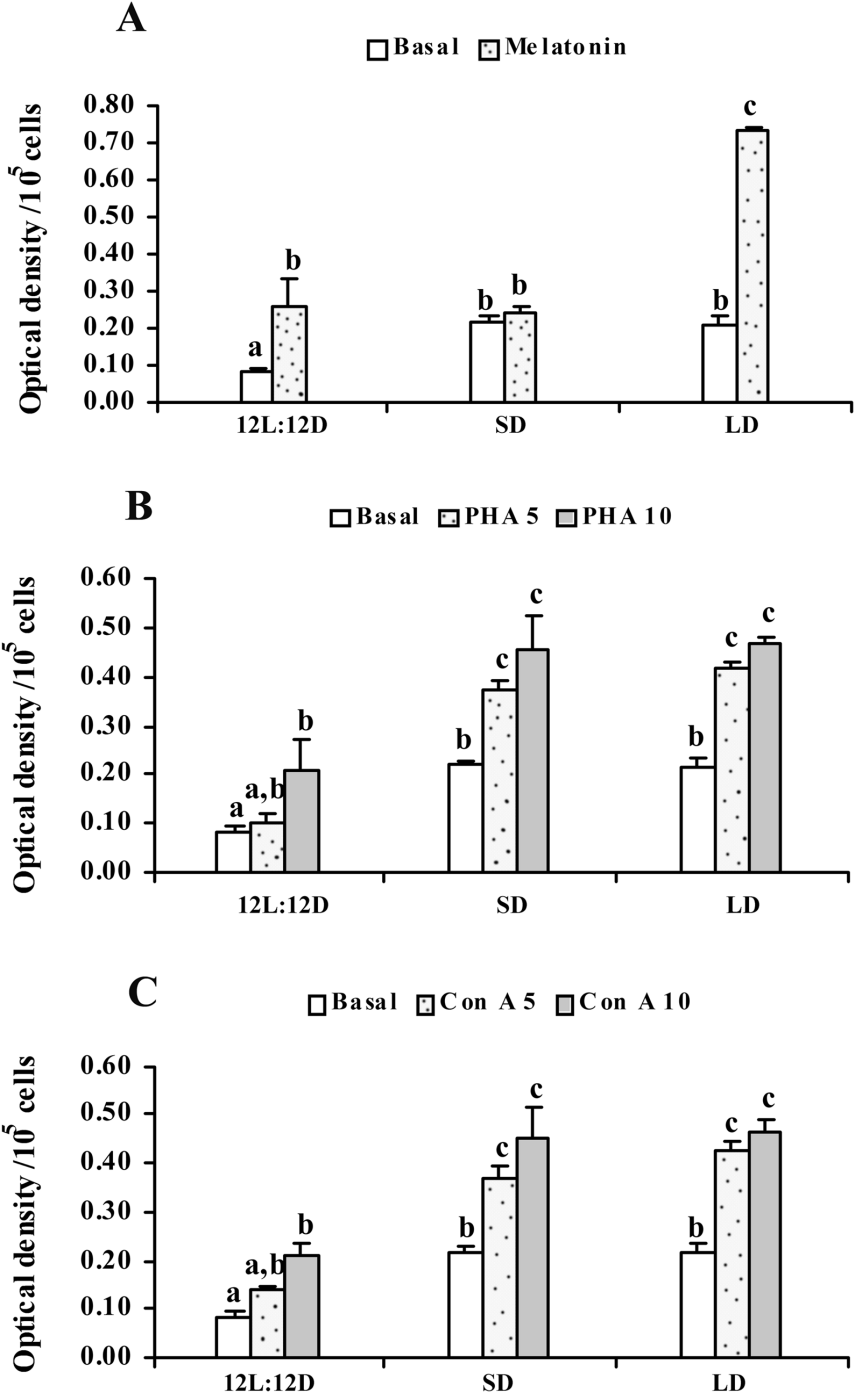
Effect of different photoperiodic regimens (12L:12D—light:dark 12:12; SD—short days, light:dark 8:16; LD—long days, light:dark 16:8) on basal **(A)** and mitogens (Con A- **(C)** and PHA- **(B)**) induced lymphocyte proliferation in the fresh-water snake, *Natrix piscator*. Effect of in vitro melatonin (500 pg/mL, upper panel) on lymphocyte proliferation is also shown. The error bars bearing the same superscript do not differ significantly (Newman–Keul’s multiple-range test, p < 0.05).

So far, phagocytosis by leucocytes is concerned, the number of cells performing phagocytosis decreased significantly (df = 27; F = 9.618 and p < 0.001) in the snakes kept in 08L:16D, however, there was no significant change in phagocytosis in animals kept in long days, when compared with control animals (Fig. 3C). Nitrite release decreased significantly (df = 38; F = 118.674 and p < 0.001) in leucocyte cultures, obtained from animals kept in long days. In contrast, nitrite release was significantly enhanced in cultures taken from snakes kept in short days, when compared with control animals (Fig. 3A). Super oxide production (NBT reduction) decreased in the leucocytes culture obtained either from short or long day animals, when compared with the control snakes (df = 26; F = 23.471 and p < 0.001) (Fig. 3B). Significant (df = 116; F = 21.695 and p < 0.001) enhancement of basal and Con A stimulated proliferation of lymphocyte was observed in cultures obtained from animals kept in short or long days (Fig. 4C). Somewhat similar trend was observed with PHA stimulated lymphocyte proliferation (df = 116; F = 19.492 and p < 0.001) where basal and PHA stimulated proliferations of lymphocyte were observed in cultures obtained from animals kept in short or long days (Fig. 4B). In vitro melatonin (500 pg ml^-1^) significantly (df = 77; F = 90.445 and p < 0.001) stimulated proliferation of the lymphocyte cultures obtained from animals kept in 16L:08D, as compared to that from animals kept in 08L:16D or 12L:12D (Fig. 4A).

## Discussion

Photoperiod is the principal cue that animals use to determine seasonality. Species in temperate zone utilize the direction of change in photoperiod to start or stop important physiological processes such as reproduction. Even in the tropics, where animals remain active throughout the year, most animals reproduce seasonally. Restriction of reproduction to specific times of the year has adaptive significance as biotic and abiotic factors drive the seasonality in reproduction^32^. We posited that photoperiod modulates the immune responses differentially in order to correspond with the changing environment. Along with the suppression of reproductive function and growth, short photoperiod is also reported to influence immune functions. In non-tropical rodents, photoperiod modulates several aspects of immunity (33). In the present study, total leucocytes, lymphocyte, heterophil and monocyte counts decreased in the snakes kept under short day conditions, however, number of basophil and eosinophils increased in animals kept in long day conditions. Earlier works have shown that the number of total leucocyte, lymphocyte, specific T-cell, and Natural Killer Cell (NK Cell) are increased in short day exposed hamsters; but monocyte, neutrophil and B-cell numbers do not differ^33–36^. Short photoperiod stimulates NK Cell mediated cytotoxicity and number of circulating T- and B-cells^37,38^. Vaughan et al.^17^ and Drazen et al.^39^ have reported significant increase in total splenocyte number, and macrophage count in hamster exposed to short photoperiod; while no difference has been detected in the total or the differential leucocyte counts in laboratory rats, exposed to long or short photoperiod^40^. Kliger et al.^19^ have shown that exposure to continuous light increases heterophil number but decreases the number of lymphocytes in chickens. Differential changes in the number of leucocytes can be explained by two ways. Earlier works^41^ suggested that melatonin can change the number of blood cells or it can alter the intrinsic mitogenic activities of lymphocytes. The results of the current study show that both have happened. First, short photoperiod has altered the leucocyte populations and second, photoperiodic manipulation has differentially affected the mitogen stimulated lymphocyte proliferations. Thus, the enhanced immune response can be attributed to increase in cellular activity or simply to an increase in percentages of certain cell types.

In contrast to our predictions, photoperiod did not alter all aspects of immune function as phagocytosis and super oxide production by leucocytes decreased in animals kept on either short or long day regimens when compared with animals kept in 12L:12D. Immunological non-responsiveness to photoperiod has also been documented in laboratory rats^40^ and in tropical rodent, Aztec mice^42^, as the leucocyte number, humoral immune response, cutaneous immune response as well as splenocyte proliferation responses are not different in rodents kept under short days when compared to rodents kept under long days. These studies have demonstrated that difference in the responses of species to photoperiod is an attribute of group and species difference. Short day induced decrease in superoxide production and phagocytosis can partly be explained by the observation of Turkowska et al.^14^. In their earlier experiments, they found that particular immune responses were differently modified by continuous lighting conditions and melatonin supplementation in birds^43^. Melatonin is not the sole factor responsible for the seasonality in immunity and hence short day induced enhancement of immunity is not always the case. On the other hand, nitrite release was increased in short day snakes, but decreased in snakes kept in long days. Similar observation was noted by Yellon et al.^37^ and Bilbo et al.^44^ where they reported decrease in oxidative burst activity, T-Cell dependent humoral immunity, phagocytosis, lymphocyte IL-1a production and in vitro lymphocyte proliferation in hamsters kept under short day conditions. However, Vaughan et al.^17^ and Drazen et al.^39^ have reported significant increase in splenic mass, total splenocyte number, macrophage count, and lymphocyte proliferation in hamster exposed to short photoperiod. Inconsistent results pertaining to the differential pattern of immune response has also been described by Brainard et al.^45^ and Zysling et al.^8^. They have shown that the number of splenic lymphocyte and splenic macrophage count are significantly elevated in short day housed Syrian hamsters when compared with animals kept in long days; whereas, humoral immunity, as assessed by serum antibody concentrations, is unaffected by changes in day lengths. In our study nitric oxide production was decreased significantly in the snakes housed in long days which might be due to reduced biosynthesis of melatonin in day light. In our previous study, we had found that melatonin, secreted predominantly during dark, plays a function in regulation of the immunity^31^. Here, we demonstrate that evening injection of melatonin causes an increase in leucocyte number in *Natrix piscator*. This observation was consistent with observations in mammal where Rai and Haldar^46^ reported low TLC (total leukocyte count) in pinealectomised Indian squirrels. In the present study, we found that T-cell mitogens (Con A and PHA) induced blood lymphocyte proliferation was enhanced in snakes kept either in short or long days. Differential regulation of immune function has also been reported in birds by Majewski et al.^47^ where ROS level and spelnocyte proliferation were higher in summer. These results are in disparity to the results described by Kliger et al.^20^ where they found non-significant change in lymphocyte proliferation due to different photoperiodic regimens in chickens. They further observed that there was no effect of different photoperiodic regimens on the lymphocytes proliferation from three-week old birds to B- and T-cell mitogens (pokeweed mitogen and Con A respectively) when the lymphocytes were cultured with melatonin. However, the authors again reported that when lymphocytes from six week old birds were cultured, mitogen stimulated lymphocytes, grown in uniform light, were significantly more reactive to pineal hormone when compared with chickens kept in either irregular or intermediate light. We also found that lymphocytes isolated from animals kept in long days were more reactive to pineal hormone stimulation than from animals kept either in 12L:12D or 16L:08D. Lighting conditions caused development of inflammation while winter like photoperiod caused virtually no inflammation in chickens^48^. Results of various studies in mammals have drawn two important evolutionary relationships between reproduction and immunity. First, long day induced typical immune responses coincide with short day pattern of gonadal maturation^49,50^. There is no known example of enhanced short day immune function and large functional gonads, so it remains unknown whether these two physiological processes are able to occur together. Second, photoperiod induced immunecompetence evolved independently of seasonal reproductive function.

Our study shows that photoperiodic manipulation influences the immune activity differentially which is of adaptive significance to the animal. This conclusion is consistent with the possible role of melatonin in the control of the diurnal and seasonal variability of bird immune defense and remains in line with the general agreement that particular immune parameters are differently regulated by melatonin^13^. *Natrix piscator* breeds seasonally and it has been suggested that in seasonally breeding animals, this seasonality permits individuals to sustain an enhanced immune status for a greater portion of the year. Since energy expenditure in obtaining optimal immune status and reproduction is highly unlikely^51–53^, natural selection has favoured energy expenditure in these processes at a specific time of the year^1^. *Natrix piscator* breeds from September to December when their gonads are maximal in size^54^ and as per previously reported hypothesis of energy trade-off between immunity and reproduction^55^, it is concluded that in wild animals elevated pineal hormone in winter suppresses reproduction while immunity is stimulated. Seasonal fluctuation in immune function is associated with the seasonal adjustment in diseases and death rates and a good correlation exists between them. Since photoperiod is primary cue to determine the season of the year, elevated status of immune function of seasonal breeders helps them fight with the seasonal challenges that will otherwise compromise immune responses and ultimately jeopardize the survival of species. It has been postulated that photoperiod induces changes in immune status which may permit adaptive functional responses in order to maintain seasonal energetic budgets of animals. Most physiological responses (like elevated immune status) are energetically expensive, therefore, animals have evolved a strategy to reduce immune function at certain times of the year when energy is diverted towards reproductive activities. However, when environmental conditions are not suitable for reproduction, investment in survival and defense (immunity) is favored.

## Materials and methods

### Animals

Male *Natrix piscator* (commonly called as fresh water snake), having weight 80–120 g, were collected during Feb and Mar from a local person who found the animals in small ponds and ditches in Varanasi (28^0^ 18′ N; 83^0^01′ E), India. The snakes were kept in the laboratory (experiencing natural ambient photoperiod and temperature) and were acclimated for four weeks before the study was started. These snakes were kept in cages (made up of wood and wire net; size 50 × 30 × 30 cm). Each cage was made up of wooden floor having sides of wire net and a window on one side. Each cage was equipped with an earthen pot of 4 L capacity, filled with water and accommodated 4–5 snakes. The animals were given small fishes as food ab libitum. Each cage was regularly cleaned and earthen pot water was changed after feeding. The regulations of the committee for the purpose of control and supervision of experiment on animals (CPCSEA), Ministry of Statistics & Programme Implementation, Government of India, were followed in the care of the animals. The experimental protocols were approved by Udai Pratap Autonomous College Ethical Committee.

### Chemicals

NBT (Nitroblue Tetrazolium salt) (Product no. N6876), MTT [3-(4, 5-dimethylthiozol-2-yl)-2, 5 diphenyltetrazolium bromide] (Product no. M2128), melatonin (Product no. M5250), and mitogens (Con A, Concanavalin A [Product no.L7647]; PHA, Phytohemagglutinin [Product no. 11082132001]) were obtained from Sigma Chemicals, Sigma-Aldrich, USA. Lymphocyte separation medium (HiSep) (Product no. LSM1084, LS003), culture medium (RPMI-1640) (Product no. AL060A), gentamycin (Product no. TC026), dimethylsulfoxide (DMSO) (Product no. MB058), fetal bovine serum (FBS) (Product no. RM1112), l-glutamine (Product no. TC243), and other chemicals were bought from Himedia Lab Pvt. Ltd., India. Culture medium was mixed with 10 µl ml^−1^ of 200 mM l-glutamine, 1 µl ml^−1^ Gentamycin, 10 µl ml^−1^ anti-anti (Gibco) and 5% FBS and was called as complete culture medium. All the chemicals purchased were of analytical grade.

### Experiment

After acclimation of the animals in laboratory for 4 weeks, experiments were started. The animals were grouped into three having six animals in each group. Group 1 animals served as control and maintained in natural photoperiod of 12L:12D. Group 2 animals were maintained in long day (LD, light:dark 16:8, lights put off at 2,000 h). Group 3 animals were maintained in short day (SD, light:dark 8:16, lights put off at 1,600 h). Animal groups were treated with different photoperiodic regimen for 30 days. After the completion of the experiments, animals were mildly anaesthetized and about 2 ml of blood was isolated from each animal through cardiocentesis in heparinized tubes. It has been reported that ethylenediamine tetraacetic acid (EDTA) causes haemolysis of blood in reptiles, heparin is preferred as an anticoagulant^56^. The blood was utilized to study the NBT slide assay, total leucocyte count, differentiation leucocyte count, quantitative NBT reduction assay, leucocyte phagocytosis, lymphocyte proliferation, and nitrite assay. Lymphocytes from different experimental animals were also incubated with melatonin and mitogen induced proliferation was studied.

### Total leucocyte count (TLC)

Turk’s solution (0.2% gentian violet solution in 3% acetic acid) was used as diluting fluid for counting white blood cells^57^. Twenty microlitres of blood was diluted with 380 µl Turk’s diluting fluid. The diluted blood was spread on Haemocytometer and white blood cells from the four chambers were counted, and the number of leucocytes in /mm^3^ was calculated.

### Differential leucocyte count (DLC)

A small drop of blood was taken on a clean dry slide and a uniform film was smeared. Slides were dried in air, fixed in methanol^58,59^ and stained in Giemsa and Leishman stain^60,61^. The slides were then washed in running water, dried, dehydrated, cleaned in xylene and mounted. The purpose of differential leucocyte count was to find out the number of individual leucocytes. The stained slides, as above, were observed under microscope (magnification 100X) from one end to other. Total hundred leucocytes were identified and counted from different areas of slide. Once the percentage of lymphocytes, monocytes, heterophils, basophils and eosinophils was obtained, their exact number was obtained in /mm^3^ blood from total leucocyte count.

### Phagocytic assay

Yeast cells were utilized as target cells to study phagocytosis. Suspension of yeast cells was made by adding 20 mg of baker’s yeast (*Saccharomyces cerevisae*) in 0.2 M PBS (10 ml). The suspension was put at 80 °C for 15 min and was cooled. Yeast cells were then washed three times in PBS and were finally mixed in complete culture medium. Cells were counted using haemocytometer and density was adjusted to 1 × 10^8^ cells ml^−1^. Twenty microlitres of blood was mixed with 20 µl of yeast cell suspension in a tube and was incubated for 30 and 60 min. After 30 and 60 min, smear was made on prewashed clean glass slides. The slides were air dried, fixed in methanol, stained with Giemsa, and were examined under microscope at 100 × magnification. From each slide 100 leucocytes showing phagocytosis were counted. The percentage phagocytosis was obtained by dividing the number of leucocytes showing phagocytosis by 100. The phagocytic index was obtained by counting the average number of yeast cells phagocytosed by single leucocyte.

### Separation of leucocytes

Leucocytes were isolated from buffy coat (the layer of leucocytes between the plasma and RBCs) by slow rotation technique as described by Keller et al.^62^. Blood was centrifuged at 42 × *g* in a swinging bucket rotor for 25 min at 8 °C. The peripheral blood leucocytes (PBL) were isolated by smoothly spinning the buffy and transporting the cells into a new tube. After centrifugation at 200× *g* for 10 min, the plasma was discarded and the pellet was gently mixed in 1 ml of complete culture medium. For eliminating any residual plasma, the cells were centrifuged again at 200× *g* for 10 min at 8 °C, the supernatant was discarded, and the cells were resuspended in culture medium. The number of viable PBLs and purity were determined via trypan blue exclusion test using a hemocytometer and experiment was further processed only when viability and purity exceeded 90%. All the techniques and counts were performed by one person to ensure consistency.

### NBT assay

Respiratory burst activity and superoxide anion production by phagocyte were examined through NBT assay^63,64^. NBT is a yellow colored, water soluble, and membrane permeable dye which is reduced to NBT-diformazan (Purple color) by superoxide. NBT assay is an important measure of respiratory burst activity and has been validated in lower vertebrates^65^. NBT assay was carried out following the method of Berger and Slapnickova^66^. Briefly, NBT solution (0.1%) was made in phosphate buffer saline (PBS) and was mixed at room temperature for 1 h. This solution was kept at 4 °C. Isolated leucocytes, as above, were counted and adjusted to 2 × 10^6^ cells ml^–1^ in complete culture medium. Trypan blue exclusion test was used to determine cell viability, which was above 95%. Leucocyte suspension (50 µl) having 1 × 10^5^ cells was mixed with 50 µl of culture medium containing NBT in culture plate from each animal in triplicates. Culture medium (100 ml) alone in triplicates served as blank. Cells were then incubated in 5% CO_2_ atmosphere at 25 °C for 2 h, centrifuged at 700× *g* in cooling centrifuge, washed thrice with PBS and fixed in methanol (70%). After fixation, 20 µl of triton X-100 (0.1%) was added in each culture well. Further, 120 µl KOH (2 M) and 140 µl DMSO were added to each well to dissolve the formazan crystals present inside the cells. Absorbance was measured with the help of ELISA plate reader at 620 nm.

### Nitrite assay

Cytotoxicity involves various effector molecules and nitric oxide (NO) plays important roles in it. Phagocytes in reptiles have been reported to produce NO which acts as cytotoxic molecule and destroys pathogen^67^. NO is produced from l-arginine by enzyme nitric oxide synthase (NOS) and is very unstable compound. Nitric oxide is degraded to other nitrogen oxides such as nitrite (NO_2_^–^) and nitrate (NO_3_^–^) commonly known as reactive nitrogen intermediate (RNI)^68^. This method was thus assayed to measure the immune cytotoxicity. Nitrite content, produced by the cells in the supernatant, was measured following the method of Ding et al.^69^ with slight modification. Hundred microlitres of leucocytes (1 × 10^6^ cells ml^–1^) were seeded to each well of culture plate. Cells were incubated at 25 °C in 5% CO_2_ atmosphere for 24 h, centrifuged at 200× *g* and supernatant was taken in another well. Equal volume of Griess reagent (1% sulfanilamide in 3 N HCl and 0.1% naphthylenediamine dihydrochloride in distilled water) and supernatant were added together and absorbance of the solution was read at 540 nm with the help of plate reader. Wells having culture medium alone without any cell served as blank. Following blank subtraction, triplicates were averaged.

### Lymphocyte proliferation assay

Lymphocyte proliferation assay is an important marker of cell mediated immune response and its role in immunity has been reviewed in reptiles by Zimmermann et al.^70^. Proliferation of lymphocytes was assessed through colorimetric assay which utilizes tetrazolium salt (MTT) as described by Berridge et al.^71^. The method, utilizing MTT salt, is an advantageous alternative method measuring proliferation of lymphocytes^72^. Tetrazolium salts enter the mitochondria of metabolically active cells and mitochondrial dehydrogenase enzyme breaks thetetrazolium rings of MTT. Tetrazolium salts are reduced into dark blue formazan crystals which remain inside the cells. Formazan crystals are liberated after solubilisation of cells by the addition of a detergent. The amount of formazan product, as measured by the amount of absorbance 570 nm is directly related to the number of cultured cells^73^. Thus, MTT assay provides a measure of cell number during last hours of in vitro culture. Collection of colored formazan products in the cells is positively correlated with the incorporation of ^3^H-thymidine into DNA in the S-Phase of cell division during last hours of in vitro culture^74^. Lymphocytes were segregated from whole blood by density gradient centrifugation using lymphocyte separation gradient HiSep having density 1.077 g ml^−1^. Blood was carefully leveled over equal volume of HiSep in a test tube and centrifuged at 400× *g* for 30 min at 8 ^o^C with brakes off. After centrifugation, ring of lymphocyte formed at the interface between medium and HiSep was carefully taken out with the help of Pasteur’s pipette, washed thrice with PBS, counted and viability was checked through trypan blue exclusion test. Viable cells which exceeded 95% were adjusted to 2 × 10^6^ cells ml^−1^ in culture medium.

Basal and mitogen (Con A and PHA) stimulated lymphocyte proliferation assays were performed. Mitogens were dissolved in 0.2 M PBS (pH 7.2) at a concentration of 1 mg ml^−1^ and further working concentrations were made in culture medium. Both the mitogens were used at concentration of 5 and 10 µg ml^−1^. Fifty microlitres of mitogen and 50 µl of cell suspension, having 2 × 10^6^ cells ml^–1^,were added to the wells of culture plate. To study basal proliferation, 1 × 10^5^cells were added into well of culture plate along with 50 µl of culture medium. Wells containing only 100 µl of culture medium, acted as blank. Further, to study the effect of in vitro melatonin, 50 µl cell suspension was added along with 50 µl of melatonin (500 pg ml^–1^, final concentration).

Plates were incubated in humidified 5% CO_2_ atmosphere for 48 h at 25 °C. Following incubation, 10 µl of MTT reagent (5 mg ml^–1^) was added to each well, and plates were again incubated for 4 h. Following incubation, plates were centrifuged at 200× *g* and the supernatant was aspirated. To solubilize the formazan crystals 100 µl of DMSO was added to each well and optical density was read at 570 nm with the help of ELISA plate reader.

### Statistical analysis

All the measurements were taken in triplicate. Each experiment was performed three times with different animals (N = 5). Reproducibility was verified and then data of one of the experiments were analysed. Data are given as mean ± SEM. Various data of one independent experiment were analysed by generalized linear model procedure and by one-way Analysis of Variance (ANOVA) using SPSS 16.0 and Post hoc comparisons were carried out using Newman-Keul’s multiple-range test. Differences were considered significant when P < 0.05.

### Ethical approval

All applicable international, national, and/or institutional guidelines for the care and use of animals were followed. This article does not contain any studies with human participants performed by any of the authors.

### Informed consent

This article does not contain any studies with human participants hence informed consent is not applicable.

## Data Availability

All the necessary data pertaining to the findings of present study are available in the manuscript.
